# Camouflaging in neurodivergent and neurotypical girls at the transition to adolescence and its relationship to mental health: A participatory methods research study

**DOI:** 10.1002/jcv2.12294

**Published:** 2024-12-17

**Authors:** Ailbhe McKinney, Sarah O’Brien, Jacqueline A. Maybin, Stella W. Y. Chan, Simone Richer, Sinead Rhodes

**Affiliations:** ^1^ Child Life and Health University of Edinburgh Edinburgh UK; ^2^ Centre for Clinical Brain Sciences University of Edinburgh Edinburgh UK; ^3^ Department of Experimental Psychology University of Oxford Oxford UK; ^4^ Florence Nightingale Faculty of Nursing, Midwifery & Palliative Care Kings College London London UK; ^5^ The Centre for Reproductive Health University of Edinburgh Edinburgh UK; ^6^ Charlie Waller Institute, School of Psychology and Clinical Language Sciences University of Reading Reading UK

**Keywords:** ADHD, adolescence, autism, camouflaging, developmental co‐ordination disorder, dyspraxia, masking, mental health, participatory research methods

## Abstract

**Background:**

Adolescent girls with diagnoses of autism, ADHD and/or developmental coordination disorder (DCD) are at higher risk for mental health problems than boys with the same diagnoses and neurotypical girls. These girls are called neurodivergent here, though neurodivergence includes a broader range of diagnoses. One possible reason for this mental health disparity could be camouflaging, a coping strategy used more by girls. Camouflaging is when the individual pretends to be neurotypical, often involving substantial effort. This study aims to understand: (a) if the use of camouflaging has started by early adolescence, (b) how components of camouflaging (assimilation, masking, and compensation) present at this age, (c) if age predicts camouflaging and (d) what is the relationship with mental health.

**Methods:**

**Participatory methods:** A co‐production team of 15 adult neurodivergent women co‐produced the project and ranked camouflaging as their most important research theme.

**Main Study:** Participants were 119 girls (70 neurodivergent, 49 neurotypical) aged 11–14 years. A transdiagnostic approach was adopted and the neurodivergent group had a diagnosis of autism, ADHD and/or DCD. Girls completed self‐report measures of camouflaging, anxiety, and depression in an online meeting with a researcher.

**Results:**

Neurodivergent and neurotypical girls presented similarly on two components of camouflaging namely masking and compensation, components related to presenting in a socially acceptable way and mimicry. Groups differed on the assimilation component, which is related to trying to fit in and involves the feelings of pretending/acting. Age had a medium effect on camouflaging with higher levels of camouflaging observed in older girls. Camouflaging scores strongly predicted anxiety and depression scores in both groups.

**Conclusions:**

The use of camouflaging, specifically assimilation, is evident in a transdiagnostic sample of 11–14 year old neurodivergent girls. Importantly, the strong relationship between camouflaging and poor mental health is present at this early age, substantiating the co‐production team's insights.

## Graphical abstract

A participatory research approach was adopted and a co‐production team of 15 neurodivergent women ranked camouflaging as their most important theme to research in the context of adolescence. Our findings show that neurodivergent girls are camouflaging more at 11–14 years of age than their neurotypical peers. Importantly, the relationship between camouflaging and poor mental health is evident at this early age, during the transition to adolescence.

**Key points**

- What's known
  - Previous research has shown higher rates of camouflaging is associated with poor mental health in adults. While anyone can camouflage, it seems women tend to do it more than men. When young girls start to camouflage and when it starts to be related to mental health is not known.
- What's new
  - The study adopted a participatory research approach and was co‐produced with 15 adult neurodivergent women. They advocated for camouflaging as the most important research theme for adolescent girls.
  - This study found neurodivergent and neurotypical girls presented similarly on two components of camouflaging, namely, masking socially inappropriate behaviours/feelings and social imitation. The neurodivergent girls differed in that they felt they were acting or pretending more often than their neurotypical peers.
  - Higher camouflaging levels were associated with more anxiety and depression symptoms for both the neurodivergent and neurotypical girls.
- What's relevant
  - By the beginning of adolescence, the distinct use of camouflaging by neurodivergent girls, relative to their neurotypical peers, is evident.
  - Importantly, the relationship between camouflaging and poor mental health is observed at this early age.
  - These findings have implications for the development of early psychoeducation interventions about camouflaging.
  - The research questions and design were informed by a priority‐setting partnership and empirical evidence, further refined by a project specific co‐production team. The end result was an ethically designed project in line with the community's priorities and perspectives.

## INTRODUCTION

Adolescents with neurodevelopmental conditions like autism, ADHD or developmental coordination disorder^1^ have higher rates of depression and anxiety than their neurotypical peers (Accardo et al., 2024; Pratt & Hill, 2011; Simonoff et al., 2008; Yoshimasu et al., 2012; Zwicker et al., 2013). Within this population, girls have higher rates of depression and anxiety than boys with the same diagnoses. Autistic adolescent girls have worse mental health than autistic boys and neurotypical girls (Oswald et al., 2016; Solomon et al., 2012). The same pattern of results is evident for girls with ADHD (Castellano‐García et al., 2022; Hinshaw et al., 2012) and girls with developmental coordination disorder (DCD) (Harrowell et al., 2017; Omer et al., 2019). This gap in mental health between girls and boys with neurodevelopmental conditions widens during adolescence (Chronis‐Tuscano et al., 2010; Horwitz et al., 2023; Murray et al., 2022). There is a need to understand why adolescent girls with neurodevelopmental conditions are a special risk group for mental health difficulties (Augustine et al., 2022; Gilbert et al., 2023) and why this gap begins or widens during adolescence.

Research has consistently shown that girls and women have worse mental health than boys and men in the general population and that this disparity widens during adolescence (Kuehner, 2017; GBD 2019 Mental Disorders Collaborators (2022); Salk et al., 2017; Wade et al., 2002). The poor mental health outcomes for adolescent girls with neurodevelopmental conditions could be because of the additive risk factors of being a girl and having a neurodevelopmental condition. There could also be intersectional risk factors, factors associated with the *specific* experience of being a girl and having a neurodevelopmental condition (Saxe, 2017). One such intersectional risk factor may be camouflaging. While boys engage in camouflaging too, extant literature has indicated girls tend to do it more than boys (Cook et al., 2021; Dean et al., 2017; Hull, Lai, et al., 2020; Lai et al., 2017; Young, 2020).

Camouflaging^2^ is the conscious or subconscious suppression of socially unacceptable behaviour and the compensation of difficulties in social interaction with the goal of being perceived as neurotypical (Hull et al., 2017; Pearson & Rose, 2023). Camouflaging can involve suppressing reactions to sensory experiences, imitating social behaviour (without always understanding the social behaviour they are imitating), and developing ways of avoiding situations or perfectionism (Sedgewick et al., 2022; Young, 2020). Camouflaging offers some benefits in a largely neurotypical world, including securing education and employment, and making connections (Zhuang et al., 2023). Although, autistic advocates highlight camouflaging is often a way to stay safe and a reaction to rejection and trauma (Pearson & Rose, 2021). Camouflaging refers to supressing your authentic self in favour of projecting an inauthentic persona which often feels uncomfortable (Pearson & Rose, 2023). It is sometimes misunderstood as a form of impression management (e.g. acting differently with friends compared to parents; in the way neurotypical adolescents would) (Pearson & Rose, 2023).

Camouflaging has been defined slightly differently in the literature, but one useful framework is Hull and colleagues' conceptualisation (Hull et al., 2017, 2019) (although this model is not exhaustive). This model maintains camouflaging has three components: masking, compensation, and assimilation. Masking involves strategies used to hide autistic characteristics or portray a non‐autistic persona (for this study, it is taken to mean portray a *neurotypical* persona). Compensation is strategies used to actively compensate for difficulties in social situations. Assimilation is strategies that reflect trying to fit in with others in social situations. This model of camouflaging is reflected in the Camouflaging Autistic Traits Questionnaire (CAT‐Q) and an adapted version of which will be used in the current study. This study aims to understand which component strategies of camouflaging neurodivergent and neurotypical adolescent girls are using at 11–14 years old.

The diagnostic criteria for Autism, ADHD, and DCD are distinct but co‐occurrence is high and symptomology is overlapping (e.g. difficulty with executive function, motor control, sensory reactivity, communication [Antshel & Russo, 2019; Coghill & Sonuga‐Barke, 2012; Kopp et al., 2010; Sumner et al., 2016]). Young people with these diagnoses lead lives with similar challenges such as social exclusion, stigma, bullying, identity problems, sleep and eating problems and low academic achievement. There are also common coping strategies (both adaptive and maladaptive) across diagnostic categories. These coping strategies develop as a reaction to similar life challenges for example, following predictable routines, substance misuse, or isolation. One such common coping mechanism is camouflaging as a reaction to stigma and rejection. This paper takes a transdiagnostic approach meaning it aims to understand the common experience of camouflaging across diagnostic categories in adolescent girls. The heterogenous sample approach reflects the real‐world diversity, complexity, and intersection of neurodevelopmental conditions (Astle et al., 2021; Embracing Complexity, 2021). The transdiagnostic approach complements the neurodiversity paradigm which maintains humans vary in their neurological make‐up and that this variability dictates the ways people process information, emphasising continuums rather than categories (Fletcher‐Watson, 2022). People with neurocognitive profiles that are more common in the population are described as “neurotypical,” while those with less common profiles, who may meet diagnostic criteria for a neurodevelopmental condition like autism, ADHD, or DCD, are described as “neurodivergent”. By comparing neurotypical and neurodivergent people, the neurodivergent experience of camouflaging can be elucidated.

Historically in neurodiversity and disability research, camouflaging has mostly been researched with autistic adults. It was first proposed as a reason for why girls and women are diagnosed less often and later than boys and men with autism and researched in this context (Bargiela et al., 2016; Begeer et al., 2013; Bölte et al., 2023; Faraone et al., 2021; Lai & Baron‐Cohen, 2015; Young, 2020). More recently, it is understood not solely as a female‐specific issue, it can apply to anyone, and is not characteristic of a ‘female presentation’ of autism (Lai et al., 2020). Research has now shifted its focus to understanding the relationship between camouflaging and mental health and well‐being.

A recent systematic review has shown camouflaging is associated with social anxiety, general anxiety, depressive symptoms, and suicidality for autistic people (Cook et al., 2021). A mixed methods review reported that the negative impact camouflaging has on mental health is due to identity conflict and confusion, exacerbated feelings of difference and inadequacy, fatigue and burnout, delayed diagnosis, lack of support, and inauthentic relationships (Zhuang et al., 2023). In light of these findings, it is surprising there is limited research on the development of camouflaging across childhood and adolescence (Cook et al., 2021; Pearson & Rose, 2021), when it starts, and when it begins to show a relationship with anxiety and depression.

Qualitative studies interviewing adolescent autistic girls, their mothers, educators or autistic adult women reflecting on their adolescence indicate autistic girls attempt to camouflage in school settings and at least some are aware they are doing it (Bargiela et al., 2016; A. Cook et al., 2017; Cridland et al., 2014, Halsall et al., 2021; Tierney et al., 2016). Parents and educators reported girls camouflaged but were often limited by their social understanding. They believed girls could handle superficial interactions but found reciprocal interactions and developing friendships difficult. The motivation to camouflage mentioned by girls, their mothers and educators are like those of adults, specifically, a desire for friendship, to fit in, and to avoid bullying or humiliation. Similar to adult accounts, these studies highlight the negative consequences experienced by girls following camouflaging such as the exhaustion, missed or late diagnosis, identity conflict, and acute anxiety. Some of this anxiety was related to the uncertainty around understanding how “successful” the camouflaging was working. The current study aims to build on these findings using validated camouflaging and mental health assessments in a larger transdiagnostic sample to understand what camouflaging strategies neurodivergent 11–14‐year‐old girls are using relative to a group of neurotypical girls.

A comparison group of neurotypical participants is not completely in line with the transdiagnostic approach as the underlying assumption is that the aim is to identity deficits by making such a comparison (Astle et al., 2021; Fletcher‐Watson, 2022). Camouflaging is measured as a continuous variable using the CAT‐Q. The CAT‐Q is not able to categorise respondents into people who camouflage and those who do not or indicate who is camouflaging at a ‘high or ‘low’ level. A comparison group of neurotypical girls is necessary to demonstrate if the neurodivergent girls have begun camouflaging to a degree which indicates distinct development. This study used an observational design to compare camouflaging in a group of neurodivergent and neurotypical girls and examine the relationship with mental health to address the following research questions.

1. Are the higher camouflaging levels evident in neurodivergent adults compared to neurotypical adults also observed in neurodivergent girls at the transition to adolescence (11–14 years old)?
2. What is the camouflaging profile of neurodivergent and neurotypical girls at this developmental stage?
3. Is age predictive of camouflaging?
4. Is the association between camouflaging and poor mental health evident in adult research also observed at the beginning of adolescence?

## MATERIALS AND METHODS

### Project's participatory research methods

#### Aims of the participatory research methods

The findings presented in this paper are part of a larger project called the “Transition To Teenager Project in Neurodivergent Girls” aimed at understanding the specific aspects of being a girl and being neurodivergent which puts this group at high risk for mental health difficulties, and why this risk increases at the transition to adolescence. Transitions were ranked as one of the most important research priorities in a James Lind Alliance Priority Setting Partnership about children with neurodevelopmental conditions (Lim et al., 2019), reflecting stakeholders lived experience with this challenging time. The aims of co‐production for the current study were to firstly, collect stakeholders' view on the question of the specific challenges of girls as they transition to adolescence; these findings can be used by other groups to inform their research. Secondly, the aim of co‐production was to co‐produce a feasible project to investigate this research question.

#### Participatory methods team

**FIGURE 1:**
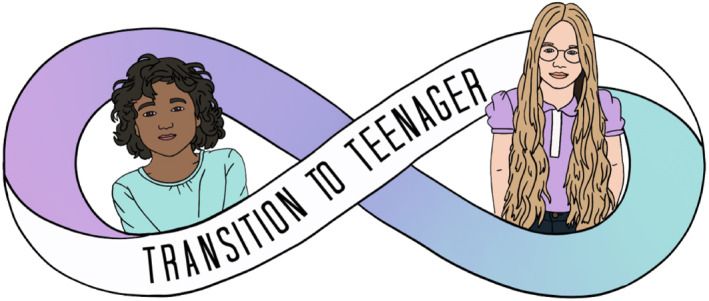
Project logo for recruitment designed by an autistic artist.

The stakeholder group was comprised of 19 neurodivergent women (age range 18–50) and girls (aged 11–13): five women with ADHD, six women with DCD, four autistic women and four adolescent neurodivergent girls. A combination of neurodivergent women with specialised skills in research or advocacy as well as women with lived experience were recruited in line with recommendations from Nicolaidis et al. (2019). Stakeholders received a £20 voucher for each hour's workshop or interview, a decision informed by the NIHR guide on payment for public involvement (National Institute for Health Research, 2022). In addition to the stakeholder group, a 25‐year‐old neurodivergent woman with relevant lived experience (autism and DCD diagnosis) and research expertise on participatory methods was recruited to the research team to be the project's co‐production co‐ordinator. She provided formative feedback at each co‐production phase, a recommendation from den Houting (2021), and was a point of contact for increasing accessibility. Finally, an autistic artist was hired to make a logo for the study to improve recruitment rates and create a sense of community (see Figure 1).

#### Framework and process of the participatory methods

**FIGURE 2:**
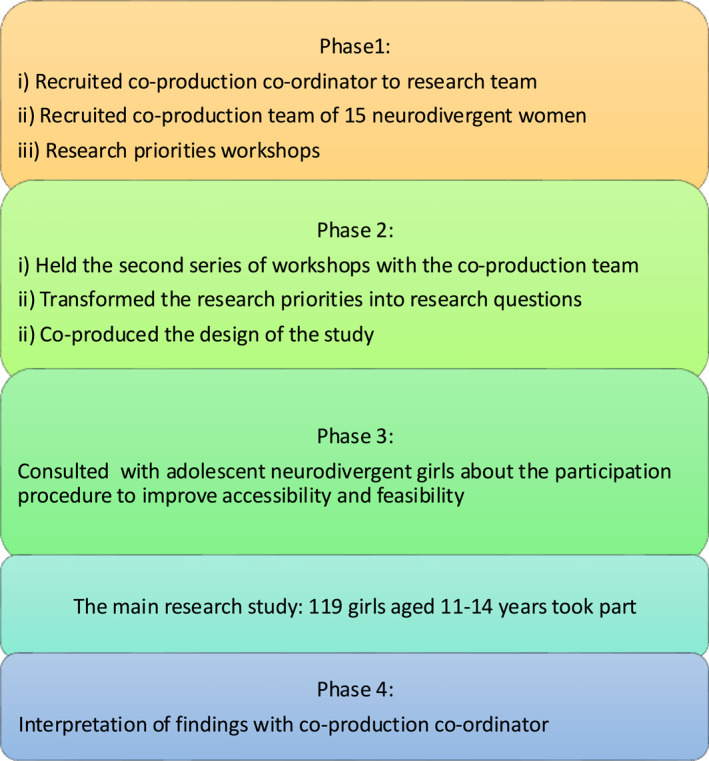
The project's research process with the participatory research approach embedded throughout.

Figure 2 shows how participatory methods were embedded throughout the research project. Practices were informed from guidelines from Nicolaidis et al. (2019) and Fletcher‐Watson et al. (2021) on conducting participatory research with neurodivergent people. Co‐production took place mostly in group workshops, but some women opted for one‐to‐one interviews or email due to different communication and access preferences. Workshops/interviews during phase 1 were held separately for the autistic, ADHD, and DCD women to explore similarities and differences in their research priorities. In an open discussion, women were asked *What are the girl specific challenges of transitioning from a child to a teenager?* Women were asked to rank the importance of the themes mentioned during the meetings in a subsequent email. The three diagnostic groups ranked camouflaging as the most important theme, thereby supporting a transdiagnostic approach for the rest of co‐production and the main research study. For the full table of research priorities ranked in order of importance please see supporting information. In Phase 2, the workshops were held with the autistic, ADHD, and DCD women together. The proposed research questions and design were presented to the women and feedback was collected, making it an iterative process. Phase 3 involved meeting with four neurodivergent adolescent girls to get feedback on the practicalities of the study protocol and to adapt the camouflaging scale. Finally, in phase 4, the co‐production co‐ordinator helped with writing the manuscript, so the perspective of a neurodivergent person was included in the interpretation of the data.

The distribution of power should be highlighted in participatory research especially in a group who have historically been marginalised. Nicolaidis et al. (2019) broadly categorised levels of involvement into three types: (a) input or consultation, (b) authentic inclusion or collaboration, or iii) an equal partnership. The co‐production team influenced key decisions about the research questions, design, and assisted with the interpretation of findings but the project was not led by community members and so the project is considered authentic collaboration.

#### Impact of participatory methods on the project

To summarise, the co‐production work impacted the project in four ways. This work (a) shaped the research questions by collecting and ranking the research priorities of stakeholders, (b) evaluated the proposed plan for the research questions and co‐produced the design of the study, (c) improved accessibility and feasibility through consultation with adolescents, and (d) collaborated with a neurodivergent co‐production co‐ordinator for the interpretation of findings. The research developed through this project is now described.

### Main research study

#### Participants

Study participants were 119 girls (neurotypical *n* = 49, neurodivergent *n* = 70 aged 11.03–14.7, *M* = 12.1, *SD* = 0.82). Recruitment was in the UK and Ireland through organisations/charities, schools, and social media. Table 1 shows the sample characteristics such as ethnicities, other conditions/diagnoses, and gender identity. The study was advertised for girls as part of a bigger study called *The Transition to Teenager in Girls Project* and participants self‐selected to take part.

**TABLE 1:** Sample characteristics.

	Total sample	Neurodivergent	Neurotypical
	119	70	49
***Ethnicity***			
White British	94	56	38
White Irish	10	7	3
White any other White background	7	5	2
Indian	2	1	1
Caribbean	1	0	1
Pakistani	1	0	1
African	1	0	1
Any other mixed or multiple ethnic background	2	1	1
Did not say	1	0	1
***Other conditions and diagnoses reported***			
Epilepsy/febrile convulsion in lifetime	4	4	0
Foetal alcohol spectrum disorders (FASD)	1	1	0
Avoidant restrictive food intake disorder (ARFID)	1	1	0
Sensory processing disorder/Sensory issues	3	3	0
Auditory processing disorder/Hyperacusis	2	2	0
Visual apraxia	1	1	0
Dyscalculia/Dysgraphia	6	6	0
Hypersensitivity (type 4)	1	1	0
‘Demand avoidance’	1	1	0
OCD	1	0	1
Tourette's syndrome/Tics	2	2	0
Hypermobility or Ehlers‐danlos syndrome	5	5	0
8p23.1 duplication syndrome	1	1	0
Asthma	2	2	0
Coeliac disease	1	1	0
Optic nerve hypoplasia	1	1	0
Noonan's syndrome	1	1	0
***Gender diverse individuals (assigned female at birth) birth)***			
Non‐binary	3	1	2
Gender not reported/experiencing gender dysphoria	2	1	1

#### Neurodivergent group

**FIGURE 3:**
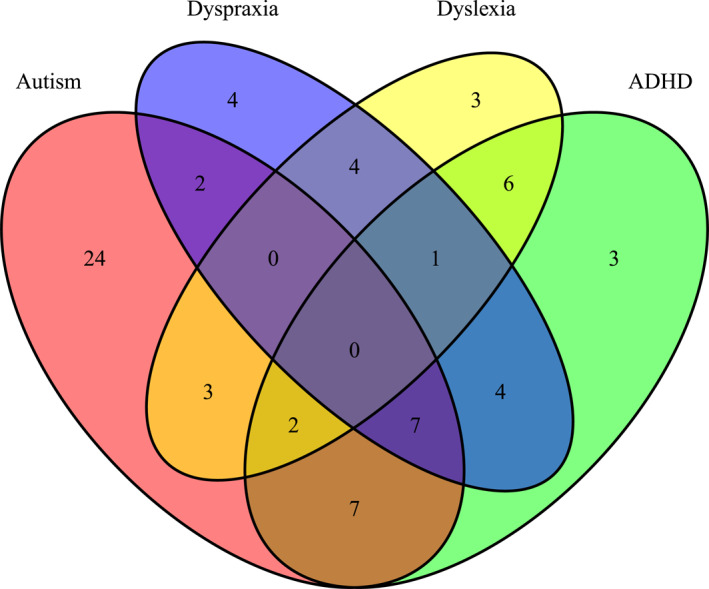
Venn diagram showing the overlap of diagnoses in the neurodivergent group (*n* = 70).

The neurodivergent group primarily had a diagnosis of autism, ADHD, DCD, dyslexia or a combination (see Figure 3). Young people on the waiting list for assessment were eligible to take part because this meant they have been referred by a GP or the child's school for a neurodevelopmental assessment and had passed an NHS Choice assessment. This assessment involves the collection and evaluation of neurodevelopmental symptom data from a parent and teacher. This allows for equitable access to research opportunities as assessment waiting lists in the public health services in the UK and Ireland are 2–4 years (British Medical Association, 2019). Fifteen adolescents on waiting lists took part (three with a diagnosis while waiting assessment for another diagnosis, and 12 who are awaiting assessment for their first diagnosis). At the point of article submission, a portion of the girls have taken part in timepoint 2 (of a two timepoint study) and so their diagnosis status was updated accordingly.

#### Neurotypical group

Table 2 shows the parent reported screening assessments of DCD, autism and ADHD collected to (a) characterise the entire sample and, (b) indicate where participants reported as neurotypical but met the cut off score on screening tools for diagnosis consideration. Of a possible total of 119, 103 parents completed the SRS‐2, 103 completed the Conners‐3 and 105 completed the DCDQ. In the neurotypical group (i.e. parents who reported their child as neurotypical) 13 participants met the cut off scores for DCD, ADHD and/or autism. These participants likely reflect a mixture of adolescents who represent broader phenotype variation and adolescents who would meet criteria for a diagnosis in a full clinical diagnostic assessment. This distinction cannot be established without further assessments and clinical judgement and, therefore all 13 were removed from the sample. Table 3 shows the mean T‐scores for each group after those who met the criteria for suspected diagnosis were removed. To investigate the research questions of this study, 106 participants were included in the analysis.

**TABLE 2:** Parent reported screening assessments.

Diagnosis	Assessment	Abbreviation	Psychometric details and properties	Cut off scores for diagnosis consideration
Developmental coordination disorder/Dyspraxia	Revised developmental coordination disorder questionnaire (Wilson et al., 2009)	DCDQ	15‐Item scale with three subscales: Control during movement, fine motor and general co‐ordination. Parents answered on a 5‐point likert scale from ‘extremely like your child’ to ‘not at all like your child’. It has demonstrated good internal reliability (*α* = 0.89).	T scores of 58 and above indicate the child probably does not have DCD and scores of 57 and below indicates the child is suspect for a DCD diagnosis
Autistic	Social responsiveness Scale‐2^nd^ edition (Constantino & Gruber, 2012)	SRS‐2	65‐Item scale with subscales: Social communication and interaction restricted interests and repetitive behaviour. Parents answered on a 4‐point likert scale from ‘not true at all’ to ‘almost always true’. It has demonstrated good internal reliability (*α* = 0.86–0.89).	T‐scores above 65 are associated with an autism diagnosis
ADHD inattentive presentation	Conners' comprehensive behaviour rating scales‐ 3^rd^ edition (Conners, 2008).	Conners‐3	110‐Item scale with subscales: Inattention, hyperactivity/Impulsivity, learning problems, executive functioning, defiance/Aggression and peer relations. Parents answered on a 4‐point likert scale from ‘not true at all’ to ‘very much true’. It has demonstrated high levels of internal consistency (*α* = 0.8–0.95).	At least 6 out of 9 DSM‐5 symptom counts are associated with a diagnosis of ADHD inattentive presentation
ADHD predominantly hyperactive‐impulsive presentation				At least 6 out of 9 DSM‐5 symptom counts are associated with a diagnosis of ADHD predominantly hyperactive‐impulsive presentation
ADHD combined presentation				At least 6 out of 9 DSM‐5 symptom counts for both inattentive presentation and predominantly hyperactive‐impulsive presentation is associated with a diagnosis of ADHD combined presentation

**TABLE 3:** Mean T‐scores by neurotype on the parent assessments of developmental coordination disorder (DCD), autism, and ADHD.

Assessment	Diagnosis	*T*‐scores	
		Neurodivergent	Neurotypical
DCDQ	Developmental co‐ordination disorder	40.89	69.7
SRS‐2	Autism	73.08	47.07
Conners‐3	ADHD predominantly inattention	75.02	48.53
Conners‐3	ADHD predominantly hyperactive‐impulsive presentation	72.46	48.8

### Procedure

The study was approved by the Edinburgh Medical School Research Ethics Committee (REC Reference: 22‐EMREC‐019). The researcher and the participant met online (Zoom or Microsoft TEAMS) so that the researcher could explain any items the participant did not understand to maintain data quality. No financial reimbursement was offered for taking part. The information sheet indicated “To take part, girls should be able to answer questions verbally with a researcher or complete questionnaires with the help of a researcher.” Written consent was taken from guardians. Demographic information (date of birth, diagnosis, ethnic group, and medication) was collected using a parent background questionnaire designed for this study. The verbal assent was ascertained at the beginning of the meeting after the procedure was explained to the adolescent. Assent was viewed as an ongoing process and participants were asked if they wanted to finish the rest of the questions two times in the meeting. The adolescent self‐report measures (Table 4) were administered on the JISC online survey platform. The researcher read out the item and the participant would tick a box on a Likert scale on their screen. The researcher explained they could not see their answers during the meeting and that their data would not be identifiable. Parent questionnaires (Table 2) were emailed to guardians after the meeting.

**TABLE 4:** Adolescent self‐report measures.

Outcome	Outcome measure	Abbreviation	Psychometric details and properties
Camouflaging	Camouflaging autistic traits questionnaire adapted for adolescents. The CAT‐Q (Hull et al., 2019) was adapted for adolescents for this study (see supporting information to read the CAT‐Q‐A).	CAT‐Q‐Adapted (CAT‐Q‐A)	25‐Item scale with three subscales: Masking, compensation, and assimilation. The CAT‐Q has demonstrated excellent internal consistency (*α* = 0.94) and acceptable test re‐test reliability (0.77) in adult populations. Participants answered on a 7‐point likert scale from ‘strongly agree’ to ‘strongly disagree’. Higher scores reflect more camouflaging. In the current study, the CAT‐Q‐Adapted had good internal consistency for the total scale (Cronbach's *α* = 0.89), and the compensation (*α* = 0.8), masking (*α* = 0.78), and assimilation (*α* = 0.77) subscales.
Anxiety and depression in children	Revised child anxiety and depression Scale–25 child report (Muris et al., 2002)	RCADS	25‐Item scale with two subscales: Depression and anxiety. Items assess the frequency of symptoms and are rated on a 4‐point likert scale from never (0) to always (3). The RCADS has shown excellent internal reliability (*α* = 0.92). Higher scores reflect more anxiety and depression symptoms. In the current study, the RCADS demonstrated excellent internal consistency (Cronbach's *α* = 0.92).
Anxiety in neurodivergent children	Anxiety scale for children—Autism spectrum disorder (Rodgers et al., 2016)	ASC‐ASD	24‐Item scale with four subscales: Performance anxiety, uncertainty, anxious arousal, and separation anxiety. The scale is an adapted version of the revised child depression and anxiety scale. Items assess the frequency of symptoms and are rated on a 4‐point likert scale from never (0) to always (3). Higher scores indicate more anxiety symptoms. In the current study, it showed excellent internal consistency (Cronbach's *α* = 0.93).

### Power analysis

A power analysis was conducted for an ANCOVA using G* power to determine the required sample size for detecting mean differences of a medium effect size (Cohen's *f* = 0.25, Partial *η* ^2^ = 0.06) with a power of 0.8, a significance level of 0.05, for two groups (neurodivergent and neurotypical) with the co‐variate of age (research questions 1–2). Based on this analysis, a minimum sample size of 128 participants group was required. This recruitment goal was not met (*N* = 106). Comparisons between the neurotypical and neurodivergent group may be underpowered to detect a difference between groups for research questions 1–2; where this is likely is highlighted below. Power analysis for a multiple linear regression conducted in G* power (research question 3–4) showed a sample of 85 was need to detect medium effect size (Cohen *f* ^2^ = 0.15) with 4 predictors (age, neurotype, camouflaging, and camouflaging*neurotype).

### Statistical analysis

All statistical analyses were conducted in R version 4.3.2 (2023‐10‐31) R Studio version 2023.12.0 + 369. To answer research questions 1 and 2 linear regression analyses were performed to test the difference in the total camouflaging scores and the subscale scores between the neurodivergent group and the neurotypical group, while controlling for age as a covariate. To answer research question 3, a linear regression was used to test if age predicts camouflaging scores. Finally, to answer research question 4, a series of hierarchical multiple linear regression analyses were conducted to examine if mental health scores (RCADS and ASC‐ASD) were predicted by camouflaging (CAT‐Q‐A) and/or neurotype (neurodivergent group or neurotypical group) while controlling for the potential influence of age. Specifically, it was tested if a model where camouflaging was included explained the difference in mental health scores between the neurotypical and neurodivergent groups compared to a model without camouflaging.

## RESULTS

**Research question 1:** Are the higher camouflaging levels evident in neurodivergent adults compared to neurotypical adults also observed in neurodivergent girls at the transition to adolescence (11–14 years old)?

Table 5 shows the mean scores of the total CAT‐Q‐A, the CAT‐Q‐A subscales and the anxiety and depression symptoms by neurotype group. A Bonferroni adjusted level to 0.0125 for four comparisons for the CAT‐Q‐A total and three subscales was used to determine significance for research question 1 and 2. Partial eta squared was used as a measure of effect size for ANCOVA (where values 0.01–0.05 = small effect, 0.6–0.14 = medium, and above 0.14 = large effect (Cohen, 2013)). There was no difference between the neurodivergent and neurotypical groups in age, *p* = 0.16, Cohen's *d* = 0.2.

**TABLE 5:** Means of age, camouflaging, and mental health scores by neurotype.

Variable	Neurodivergent (*n* = 70)	Neurotypical (*n* = 36)	*p*	Partial *η* ^2^
Age	12.2 (0.9)	12.0 (0.7)	0.16	
CAT‐Q‐A total	106.7 (24.5)	96.22 (20.59)	0.06	0.05
Masking	39.49 (9.37)	39.2 (6.84)	0.82	∼0
Compensation	33 (10.34)	27.92 (9.12)	0.02	0.06
Assimilation	34.23 (8.89)	29.11 (8.84)	0.01	0.07
ASC‐ASD	33.7 (15.2)	26 (11.8)	0.01	0.06
RCADS	29.7 (13.9)	23.5 (12)	0.04	0.05

*Note*: Standard deviations are in parentheses. A Bonferroni adjusted level to 0.0125 for four comparisons for the CAT‐Q‐A subscales (three subscales and total scale) was used to determine significance. These *p* values and partial *η* ^*2*^ are after controlling for age.

To address research question 1, a linear regression was used to investigate the impact of neurotype (neurodivergent or neurotypical) on the total CAT‐Q‐A scores, while controlling for age. The regression analysis showed a statistically significant overall model *F(*2, 103) = 5.71, *p* = 0.004, but neurotype did not show an effect on the CAT‐Q‐A total scores, *p* = 0.06. Age was a significant covariate of CAT‐Q‐A scores (*p* = 0.013), more detail about this finding where research question 3 is discussed. In summary, there was no difference in the total camouflaging scores between the neurodivergent and neurotypical girls.

**Research question 2** **:** What is the camouflaging profile of neurodivergent and neurotypical girls at this developmental stage?

To further explore if camouflaging was different between groups, the subscales were investigated. Table 5 shows the mean scores for the subscales. Three linear regressions were used to examine the relationship between the CAT‐Q‐A subscales and neurotype, while controlling for age.

Masking subscale: The overall model for the masking subscale was significant, *F*(2, 103) = 5.007, *p* = 0.008, driven by the effect of age (*β* = 0.3, *p* = 0.002). There was no effect of neurotype group on the masking subscale score (*p* = 0.82).

Compensation subscale: The overall model for the compensation subscale was significant *F*(2, 103) = 3.7, *p* = 0.028, with the neurotype predicting compensation scores (*β* = −0.48, *p* = 0.022) and age showing no effect (*p* = 0.27) after adjusting alpha using Bonferroni corrections. After adjusting the alpha level to *α* = 0.0125, the effect of neurotype was no longer significant, although approaching, *p* = 0.022. With a larger sample, this may have reached significance given the medium effect size partial *η* ^2^ = 0.06.

Assimilation subscale: The model for the assimilation subscale was significant *F* (2, 103) = 6.80, *p* = 0.002. Neurotype (*β* = −0.5, *p* = 0.012) and age (*β* = 0.22, *p* = 0.023) predicted the score on assimilation subscale with a medium effect size, partial *η* ^2^ = 0.07.

To summarise for research question 2, the neurodivergent and neurotypical girls scored similarly on two subscales of camouflaging (masking and compensation) but the neurodivergent girls scored higher on the assimilation subscale.

**Research question 3:** Is age predictive of camouflaging?

**FIGURE 4:**
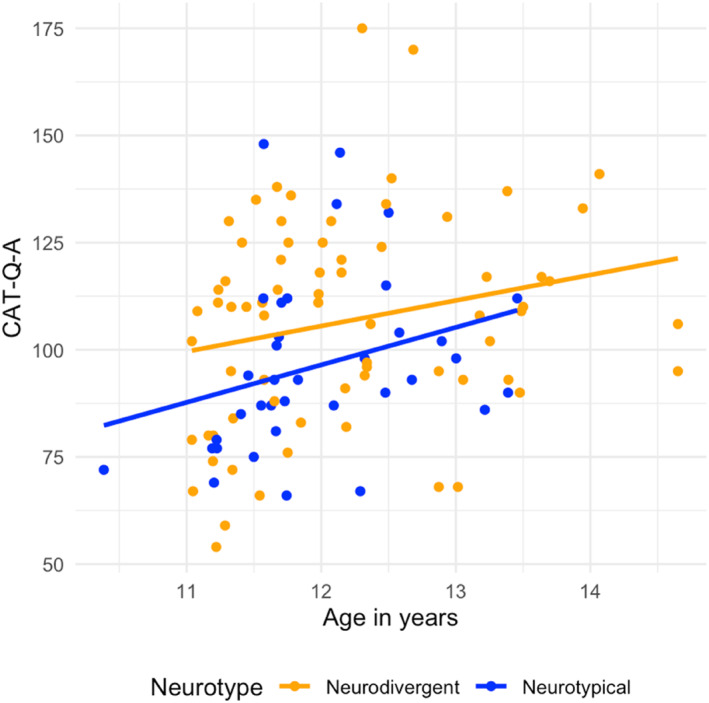
Association between camouflaging scores measured by the CAT‐Q‐A and age in years. Each dot represents an individual participant.

Figure 4 shows the results from research question 3, which aimed to investigate if age was predictive of camouflaging scores. The regression analysis showed a statistically significant overall model *F*(2, 103) = 5.71, *p* = 0.004, and neurotype did not show an effect on the total CAT‐Q‐A scores (*p* = 0.057), as discussed in research question 1. Age was a significant predictor of the total CAT‐Q‐A scores with a medium effect size, (*β* = 0.24, *B* = 6.6, *p* = 0.013, partial *η* ^*2*^ = 0.06). For a 1 year increase, camouflaging increased by 6.6 points on the total CAT‐Q‐A across neurotypes, see Figure 4. Over a short age range (11–14 years), age predicted camouflaging scores, with the older girls scoring higher than the younger girls.

**Research question 4:** Is the association between camouflaging and poor mental health evident in adult research also observed at the beginning of adolescence?

Firstly, the relationship between the CAT‐Q‐A and anxiety measured by the ASC‐ASD was investigated and secondly, the relationship between depression and anxiety measured by the RCADS was examined.

**Camouflaging and anxiety scores**

In step 1 of the model, the ASC‐ASD scores were the outcome variable, and age and neurotype were the predictor variables (see Table 6). The regression equation was significant *F*(2, 103) = 0.4.03, *p* = 0.02, with an adjusted *R* ^2^ of 0.05. Neurotype significantly predicted ASC‐ASD scores (*β* = −0.5, *p* = 0.01) but not age (*p* = 0.33).

**TABLE 6:** Hierarchical regression results for anxiety symptoms measured by ASC‐ASD.

	Variable	B	SE B	β	*p*	DF	*F*	*p*	*R* ^2^ Adj
Step 1 model						(2, 103)	4.03	0.02	0.05
	Age	1.59	1.62	0.09	0.33				
	Neurotype	−7.37	2.92	−0.5	0.01				
Step 2 model						(3, 102)	37.82	<0.001	0.5
	Age	−1.27	1.20	−0.07	0.29				
	Neurotype	−3.44	2.15	−0.24	0.11				
	CAT‐Q‐A	0.44	0.04	0.71	<0.001				
Step 3 model						(4, 101)	28.14	<0.001	0.51
	Age	−1.27	1.21	−0.07	0.3				
	Neurotype	−0.3	9.90	−0.24	0.98				
	CAT‐Q‐A	0.44	0.05	0.72	<0.001				
	Neurotype: CAT‐Q	−0.03	0.1	−0.05	0.75				

In step 2 of the model, CAT‐Q‐A is added as a predictor variable. The regression equation was significant *F*(2, 102) = 37.82, *p* < 0.001 and the adjusted *R* ^2^ increased to 0.5. CAT‐Q‐A significantly predicted the ASC‐ASD scores (*β* = 0.71, *p* < 0.0001) and the effect of neurotype was eliminated (*β* = −0.24, *p* = 0.11). Model 1 and model 2 were significantly different *F* = 97.82, *p* < 0.001.

In step 3 of the model, the interaction of CAT‐Q‐A and neurotype was explored but no interaction effect was observed (*p* = 0.75) and there was no significant difference between model 2 and 3, *F* = 0.1, *p* = 0.75.

**Camouflaging and anxiety and depression scores**

In model 1, the RCADS scores was the outcome variable and age and neurotype the were the predictor variables (see Table 7). The regression equation was significant *F*(2, 101) = 4.87, *p* < 0.001, with an adjusted *R* ^2^ of 0.07. RCADS scores were significantly predicted by neurotype (*β* = −0.4, *p* = 0.047) and age (*β* = 0.2, *p* = 0.04).

**TABLE 7:** Hierarchical regression results for depression and anxiety symptoms measured by RCADS.

	Variable	B	SE B	β	*p*	DF	*F*	*p*	*R* ^2^ Adj
Step 1 model						(2, 101)	4.87	<0.001	0.07
	Age	3.184	1.501	0.2	0.04				
	Neurotype	−5.46	2.71	−0.4	0.047				
Step 2 model						(3, 100)	43.7	<0.001	0.55
	Age	0.46	1.07	0.03	0.67				
	Neurotype	−1.8	1.91	−0.13	0.35				
	CAT‐Q‐A	0.41	0.04	0.72	<0.001				
Step 3 model						(4, 99)	32.53	<0.001	0.55
	Age	0.46	1.08	0.03	0.67				
	Neurotype	1.52	8.82	−0.14	0.86				
	CAT‐Q‐A	0.42	0.05	0.74	<0.001				
	Neurotype:CAT‐Q‐A	−0.03	0.087	−0.06	0.7				

In step 2 of the model, CAT‐Q‐A is added as a predictor variable. The regression equation was significant *F*(3, 100) = 43.7, *p* < 0.001 and the adjusted *R* ^2^ increased to 0.55. CAT‐Q‐A significantly predicted the RCADS scores (*β* = 0.72, *p* < 0.001) and the effect of neurotype and age was eliminated (*β* = −0.13, *p* = 0.35; *β* = 0.03, *p* = 0.67). Model 1 and model 2 were significantly different *F* = 110.78, *p* < 0.001.

In step 3 of the model, the interaction of CAT‐Q‐A and neurotype was explored but no interaction effect was observed (*p* = 0.7) and there was no significant difference between model 2 and 3, *F* = 0.15, *p* = 0.7.

In brief, camouflaging scores predicted higher (worse) anxiety and depression symptoms using both mental health questionnaires for neurodivergent and neurotypical girls.

## DISCUSSION

Adolescent girls with diagnoses of autism, ADHD and/or DCD (neurodivergent) are at higher risk for developing anxiety and depression than boys with the same diagnoses, and girls without these diagnoses. One specific factor related to being neurodivergent and a girl which may explain part of this gender gap is camouflaging. This study is the first to explore the profile of camouflaging in adolescent girls and the relationship with mental health in a transdiagnostic sample of neurodivergent and neurotypical girls. The neurodivergent and neurotypical girls presented the same on the masking and compensation components of camouflaging but differed on the assimilation component. Age predicted camouflaging, with higher levels of camouflaging observed in the older girls. Camouflaging scores significantly predicted anxiety and depression symptoms. The difference in mental health scores between the neurodivergent group and the neurotypical group was diminished when camouflaging was added to the model, suggesting this could be a factor driving the difference in mental health scores between them.

The neurodivergent and neurotypical girls presented the same on the masking and compensation components of camouflaging. Both neurodivergent and neurotypical girls are similarly worried about acting socially appropriate and learning through imitation, common experiences during adolescence, a time of great change in the self‐concept (Sebastian et al., 2008). The neurodivergent girls differed however with regards to the assimilation strategy which is related to *pretending or acting* in order to fit in. Already by the beginning of adolescence, the distinct use of camouflaging by neurodivergent girls is evident. This finding builds on a growing body of research which shows camouflaging starts early in life (Halsall et al., 2021; Howe et al., 2023). Camouflaging was originally proposed as a reason for why neurodivergent women are diagnosed later in life (Bargiela et al., 2016). Cook et al. (2021) have pointed out that most previous camouflaging research with adults has been conducted with women who were diagnosed later in life. Our findings demonstrate that camouflaging is a concern for girls diagnosed early in life and not only a feature characteristic of later diagnosed women.

Age had a medium significant predictive effect on camouflaging with older girls camouflaging more than younger girls. Two studies investigated whether age predicted camouflaging in autistic 13–18 year olds (Hull, Petrides, & Mandy, 2020; Jorgenson et al., 2020) and reported no effect. In a study with a larger age range (4–17 years; Ross et al., 2023), age predicted camouflaging with an older adolescent group (13–17 years) camouflaging more than a childhood group (4–13 years). It is noteworthy to point out that increases in self‐awareness may in part underlie increases in camouflaging with age (see Williams, 2022 for a related point). Longitudinal studies are required to delineate developmental trajectories and their influencing factors although the development of camouflaging likely depends on a child's interpersonal experiences (Loo et al., 2023; Pearson & Rose, 2021).

The association between camouflaging and poor mental health is present during early adolescence echoing adult research (Cassidy et al., 2020; Cook et al., 2021; Hull et al., 2021). An association between camouflaging and poor mental health was also reported in a similar study using self‐report measures in 13–18 year olds (Bernardin et al., 2021). This association is also reported in studies using the discrepancy method (Livingston et al., 2019; Ross et al., 2023) but was not evident in one study (Corbett et al., 2021) (see Lai et al., 2017 for more information on the discrepancy method). Findings align with qualitative research that have emphasised the adverse impact of camouflaging (Cage & Troxell‐Whitman, 2019; Chapman et al., 2022; Halsall et al., 2021; Rhodes et al., 2023). Cross‐sectional studies cannot establish direction of the relationship, but qualitative research and this project's co‐production team support the idea that camouflaging contributes to poor mental health. Separating the initial anxiety which instigated camouflaging, from the anxiety which came about because of long‐term camouflaging, is a methodological challenge.

This study builds on the large body of work highlighting the specific experiences and challenges of being a neurodivergent adolescent girl such as difficult menstruation and sex specific puberty issues (Cridland et al., 2014; Cummins et al., 2020; Steward et al., 2018), having a “boy condition” (Cridland et al., 2014), hormonal influences on attention (Nussbaum, 2012), sexual exploitation and vulnerabilities (Bargiela et al., 2016), and later diagnosis (Bölte et al., 2023). The current study did not have a comparison group of boys with neurodevelopmental conditions (or neurotypical boys) so this work cannot show camouflaging distinctly predicts anxiety and depression symptoms in neurodivergent girls. Future work should continue to compare boys and girls, with and without neurodevelopmental conditions to further elucidate the gender specific developmental patterns of mental health difficulties, as others have done (Augustine et al., 2022; Horwitz et al., 2023; Murray et al., 2022).

### Strengths and limitations

A strength of this study was the use of measures designed for neurodivergent young people. The ASC‐ASD, adapted from the RCADS, was designed to accommodate for the confounded presentation of anxiety and autism in young people (Rodgers et al., 2016). A possible weakness is that it was designed for autistic young people, not all neurodivergent young people, although the combination of the ASC‐ASD and the RCADS was used to account for this limitation. Secondly, the CAT‐Q was adapted for this study for the target age group but was originally designed to measure camouflaging in autistic people. While there is a large overlap in diagnostic categories (Astle et al., 2021) and camouflaging is a reaction to stigma as a minority group rather than an ‘autistic’ coping mechanism, it is possible there were types of camouflaging more pertinent to ADHD or DCD which were not captured.

A key strength of this study was the genuine participatory methods approach which incorporated the voices of neurodivergent women in decisions about research questions and study design. This study offers an example of constructing research questions based on systematic, published priority‐setting partnerships (Lim et al., 2019) and empirical evidence, further refined by a project‐specific co‐production team. This approach contributed to an ethically designed project aligned with the community's priorities. Secondly, feedback on the study protocol from both neurodivergent adult women and adolescent girls likely improved the recruitment and retention rates, data quality, and minimised stress associated with participation. Finally, since research shapes societal attitudes towards neurodivergent individuals, embedding a participatory methods approach throughout the project ensured the narratives about camouflaging in neurodivergent girls and women were shaped by their own perspectives (Woods et al., 2018).

### Practical implications and future directions

Next phases of camouflaging research should involve evaluation of whether psychoeducation about camouflaging can improve young people's mental health. For example, self‐help resources (Belcher, 2022) and adaptations to therapy (Lei et al., 2024) to reduce the negative impact of camouflaging have been developed with the aim to change clinical practice. The onus should not solely be on neurodivergent people to learn to cope though. Neurotypical people could facilitate by greater acceptance and understanding of different communication styles (Cook et al., 2023). To reduce the need for camouflaging and its negative impact, the effect of psychoeducation of *both* neurotypes merits further research.

### Conclusion

This project's co‐production team of adult neurodivergent women advocated for camouflaging as the most important theme to investigate as a predictor of mental health in adolescent neurodivergent girls. Importantly they emphasised its negative impact on their own mental health when they were adolescents. This study shows, that at the beginning of adolescence, neurodivergent girls feel like they are pretending or acting more than their neurotypical peers. Previous research has shown camouflaging is associated with poor well‐being in adults. This current study builds on this finding and we demonstrate that an association with anxiety and depression is already evident at the transition to adolescence, substantiating and validating the co‐production team's experiences and insights.

## AUTHOR CONTRIBUTIONS

**Ailbhe McKinney**: Conceptualization; Data curation; Formal analysis; Investigation; Methodology; Project administration; Visualization; Writing ‐ original draft; Writing ‐ review & editing. **Sarah O'Brien**: Conceptualization; Methodology; Writing ‐ review & editing. **Jacqueline A. Maybin**: Funding acquisition; Methodology; Supervision; Writing ‐ review & editing. **Stella W. Y. Chan**: Conceptualization; Funding acquisition; Supervision. **Simone Richer**: Data curation; Project administration; Writing ‐ review & editing. **Sinead Rhodes**: Conceptualization; Funding acquisition; Investigation; Methodology; Project administration; Supervision; Writing ‐ review & editing.

## CONFLICT OF INTEREST STATEMENT

The authors declare no conflicts of interest.

## ETHICAL CONSIDERATIONS

This research was carried out at The University of Edinburgh. The study was approved by the Edinburgh Medical School Research Ethics Committee (REC Reference: 22‐EMREC‐019).

## Supporting information

Supplementary Material

## Data Availability

The unidentifiable data will be made available upon request.

## ENDNOTES

^1^
Developmental coordination disorder (DCD) is also called dyspraxia. This paper uses DCD in line with the assessment used: Revised Developmental Coordination Disorder Questionnaire (Wilson et al., 2009).

^2^
This paper uses “camouflaging” in line with terminology from Hull et al. (2019) as we use their scale in this study. It is often referred to as “masking”, “adaptive morphing”, “compensation”, and “self‐monitoring”(Pearson & Rose, 2023, p. 3).
