# Supplementary material for: Camouflaging in neurodivergent and neurotypical girls at the transition to adolescence and its relationship to mental health: A participatory methods research study
Source: JCPP Adv. 2024 Dec 17;4(4):e12294. doi: 10.1002/jcv2.12294 (PMC11669776; doi:10.1002/jcv2.12294)
Supplement: Supplementary file 1 — Supplementary Material [file JCV2-4-e12294-s001.docx]

**Supporting Information**

**Table S1.**

*Findings from phase 1 of co-production: research priorities ranked in order of importance*

| Research priorities | Importance ranking |
| --- | --- |
| Camouflaging | 1 |
| Friendships, friendships breaking down and “fitting in” | 2 |
| Puberty, hormonal changes, anxiety about body changing | 3 |
| Lack of support from school and lack of understanding from school and adults | 4 |
| Pressure to be feminine and gender expectations | 5 |
| Negative experiences with periods (pain, low mood, sensory issues) and some neurodivergent traits exasperated while on period | 6 |
| Struggling with sense of identity and self-acceptance | 7 |
| Self-esteem, comparing to other girls, body image issues | 8 |
| Bullying | 9 |
| Academic pressure | 10 |
| Sensory issues | 11 |
| Fine and gross motor skills | 12 |
| Substance misuse | 13 |

**Appendix S1.** Adapted version of the camouflaging autistic traits questionnaire.

This questionnaire is an adapted version of the Camouflaging Autistic Traits Questionnaire (CAT-Q;Hull et al., 2019). It was adapted so it would be acceptable to adolescents. Four girls (aged 11.5, 13, 13, 14) were consulted about the phrasing of the questions. Examples were taken from *Autism and Masking – how and why people do it, and the impact it can have* by Felicity Sedgewick, Laura Hull and Helen Ellis. In the CAT-Q items 3, 12, 19, 22, and 24 are reversed scored. In this adapted version, only 19 is reversed scored. Items with an asterisk (*) should be reverse scored.

Compensation = 1, 4, 5, 8, 11, 14, 17, 20, 23

Masking = 2, 6, 9, 12, 15, 18, 21, 24

Assimilation = 3, 7, 10, 13, 16, 19, 22, 25

|  | Original wording from Hull and colleagues (2019) | Adapted wording for the current study |
| --- | --- | --- |
| 1 | When I am interacting with someone, I deliberately copy their body language or facial expressions | Note: *first check if participant knows what facial expression means*  When I am talking to someone, I copy their facial expressions. Example: If someone is smiling, I smile as well even though I don’t feel like smiling |
| 2 | I monitor my body language or facial expressions so that I appear relaxed | I try to look like I am relaxed even when I’m nervous |
| 3 | I rarely feel the need to put on an act in order to get through a social situation* | When I am talking to people, I feel like I’m acting or pretending. Example: when I am talking to people, I act like somebody else, like another girl in my class. |
| 4 | I have developed a script to follow in social situations (for example, a list of questions or topics of conversation) | I plan what to say to people before I talk to them. Example: I know what questions I will ask before I start talking to someone. |
| 5 | I will repeat phrases that I have heard others say in the exact same way that I first heard them. | I try to talk *in the same way* as other people. Example: if someone is talking fast, I talk fast too. |
| 6 | I adjust my body language or facial expressions so that I appear interested by the person I am interacting with | I try to look like I am interested in the person I am talking to. Example: I smile even though someone is talking about something boring. |
| 7 | In social situations, I feel like I’m ‘performing’ rather than being myself | I act like somebody else and not myself. Example: Pretending to be like someone else or a character from a movie |
| 8 | In my own social interactions, I use behaviours that I have learned from watching other people interacting | I try to copy how other people talk |
| 9 | I always think about the impression I make on other people | I always think about what other people are thinking about me |
| 10 | I need the support of other people in order to socialise | If my friend is with me, I find it easier to talk to other people |
| 11 | I practice my facial expressions and body language to make sure they look natural | I practice doing facial expressions in mirror, so they look like everyone else’s |
| 12 | I don’t feel the need to make eye contact with other people if I don’t want to* | I feel like I have to look people in the eye even though I don’t want to |
| 13 | I have to force myself to interact with people when I am in social situations | I make myself talk to people even though I don’t want to. Example: I talk to people because they talk to me, even though I don’t want to |
| 14 | I have tried to improve my understanding of social skills by watching other people | I notice what people in my school say and copy them |
| 15 | I monitor my body language or facial expressions so that I appear interested by the person I am interacting with | I try to look like I am interested in the person I am talking to |
| 16 | When in social situations, I try to find ways to avoid interacting with others | I try to find ways of avoiding talking to people. Example: pretending to be busy on my phone |
| 17 | I have researched the rules of social interactions (for example, by studying psychology or reading books on human behaviour) to improve my own social skills | I try to learn how to talk to people by watching tv, YouTube or Tik Tok |
| 18 | I am always aware of the impression I make on other people | I always think about what other people think of me |
| 19 | I feel free to be myself when I am with other people* | I am myself when I with other people. Example: I talk about my interests around other people* |
| 20 | I learn how people use their bodies and faces to interact by watching television or films, or by reading fiction | I learn how to talk to people by watching tv, YouTube, or Tik Tok |
| 21 | I adjust my body language or facial expressions so that I appear relaxed | I try to look relaxed when I am talking to someone |
| 22 | When talking to other people, I feel like the conversation flows naturally* | Talking to people is hard work for me |
| 23 | I have spent time learning social skills from television shows and films, and try to use these in my interactions | I try to learn how to talk and what to say from watching tv or YouTube, or reading books |
| 24 | In social interactions, I do not pay attention to what my face or body are doing* | When I am talking to someone, I think about my facial expressions |
| 25 | In social situations, I feel like I am pretending to be ‘normal’ | When I am with other people, I feel like I am pretending to be like everyone else |
